# Complete Genome Sequence of *Alteromonas* Virus vB_AspP-H4/4

**DOI:** 10.1128/genomeA.00914-17

**Published:** 2017-10-26

**Authors:** René Kallies, Bärbel Kiesel, Jakob Zopfi, Lukas Y. Wick, Antonis Chatzinotas

**Affiliations:** a Department of Environmental Microbiology, Helmholtz Centre for Environmental Research–UFZ, Leipzig, Germany; b Department of Environmental Sciences, Aquatic and Stable Isotope Biogeochemistry, University of Basel, Basel, Switzerland; c German Centre for Integrative Biodiversity Research (iDiv) Halle-Jena-Leipzig, Leipzig, Germany

## Abstract

*Alteromonas* virus vB_AspP-H4/4 is a member of the *Podoviridae* family and was isolated from North Sea water in the 1970s. The complete double-stranded DNA genome has 47,631 bp with 49 predicted genes.

## GENOME ANNOUNCEMENT

*Alteromonas* virus vB_AspP-H4/4 was isolated from North Sea water collected near the island of Helgoland between 1976 and 1978 (1). It has been identified by electron microscopy as a member of the *Podoviridae* family (2) and has been used as tracer for water/colloid transport in surface waters and porous media (3). The bacterial host belongs to the genus *Alteromonas*, as determined by sequence analysis of the 16S rRNA gene (GenBank accession no. MF185399). *Alteromonas* is a genus that belongs to the phylum *Proteobacteria*, which are frequently found in sea water (4). Only a few *Alteromonas* virus genome sequences are known. The availability of further sequences should therefore help in understanding the ecology and evolution of *Alteromonas* viruses.

*Alteromonas* virus vB_AspP-H4/4 was propagated on its host, producing variably sized clear plaques. *Alteromonas* virus vB_AspP-H4/4 has an icosahedral capsid (diameter [*d*] = 41 ± 1 nm) with a short tail (length [*l*] = 6.6 nm). Plaque purification was followed by DNA preparation (5) and sequencing on an Illumina MiSeq platform, resulting in 727,086 150-bp paired-end sequencing reads. Quality-trimmed reads were assembled with SPAdes (6) and Geneious R9 to produce a single contig with a 1,540-fold coverage. Genes were predicted with Glimmer (7), Rapid Annotation using Subsystems Technology (RAST) (8), and GeneMark.hmm (9). Functions of proteins were predicted using protein (PSI) BLAST (10), HMMER (11), and the Conserved Domains Database (12). No tRNAs were found with ARAGORN (http://130.235.46.10/ARAGORN).

No close relative was identified by BLASTn analysis. However, phylogenetic analyses of three core genes (DNA polymerase, major capsid protein, and DNA maturation protein) showed similarity to *Rhizobium* phages RHEph02 (GenBank accession no. JX483874) and RHEph08 (GenBank accession no. JX483879) (42 to 47% identity at 87 to 99% coverage), two podoviruses that were isolated from rhizosphere soil samples on *Rhizobium etli* (13). Pairwise alignments of *Alteromonas* virus vB_AspP-H4/4 with these two viruses resulted in 37.17% (RHEph02) and 38.27% (RHEph08) nucleotide identities over the whole genome.

The 47,631-bp double-stranded DNA genome had a G+C content of 40.8% and a noncoding direct terminal repeat of 217 bp, based on the occurrence of a double-coverage region in the assembled contig (14). The *Alteromonas* virus vB_AspP-H4/4 genome had 49 predicted putative coding sequences and a T7 virus supergroup-like head-neck-tail module (15). The coding sequences occupied 95.84% of the genome and ranged in size from 141 to 5,124 bp. Twenty-nine coding genes were assigned to putative protein functions. Among these, 10 structural and assembly proteins were identified, including a major capsid protein, tail tubular proteins, internal virion proteins, tail fiber proteins, a protease, and terminase small and large subunits. DNA replication proteins included a DNA polymerase, DNA primase, and a DnaB-like helicase. Eight proteins involved in nucleic acid metabolism and transcription were identified, such as thymidylate synthase, ribonucleotide reductase, exonuclease, and two DNA-dependent RNA polymerases. A putative slippery sequence was identified in the two overlapping genes 39 and 40, which code for endolysin and an internal virion protein, respectively, with the latter produced from a −1 translational frameshift.

### Accession number(s).

The complete genome sequence of *Alteromonas* virus vB_AspP-H4/4 has been deposited in GenBank under accession no. MF278336.
